# The Relationship between Metacognitive Beliefs with Clinical Belongingness and Resilience among Novice Nurses in Neonatal Intensive Care Units

**DOI:** 10.1155/2023/2949772

**Published:** 2023-06-20

**Authors:** Mitra Soltanian, Rasool Payegozar, Maryam Paran, Nasrin Sharifi

**Affiliations:** ^1^Department of Nursing, School of Nursing and Midwifery, Shiraz University of Medical Sciences, Shiraz, Iran; ^2^Student Research Committee, Shiraz University of Medical Sciences, Shiraz, Iran; ^3^Department of Epidemiology, School of Nursing and Midwifery, Shiraz University of Medical Sciences, Shiraz, Iran

## Abstract

**Background:**

The neonatal intensive care unit (NICU) is a sensitive ward for nurses. However, the low nurse-to-patient ratio has led to the hiring of novice nurses into the critical areas such as NICUs. These nurses are in need of help in the clinical environment as they have no much experience caring the neonates in the real clinical setting. Therefore, it is necessary to address the individual and psychological capacities with the help of which a person can overcome the difficult situations. This study aimed to investigate the relationship between metacognitions, clinical belongingness, and resilience of novice nursing staff in NICU wards.

**Method:**

This study is a descriptive-analytical study, and the research samples were composed of 78 novice nursing staff of Neonatal Intensive Care Units from teaching hospitals. Samples were selected via a purposive sampling method. Research tools included demographic, Wells and Hatton metacognitive beliefs, Jones Levitt belonging, and Connor–Davidson resilience questionnaires. SPSS 22 software was used for data analysis.

**Results:**

The mean score of metacognitive beliefs in novice nursing staff was 92.67 ± 13.69, and it was 116.69 ± 19.11 for belongingness and 78.78 ± 14.73 for resilience. There is positive and significant relationship between metacognitive beliefs and belongingness (*p* < 0.019, *r* = 0.265). In addition, the relationship between metacognitive beliefs and resilience in novice nursing staff was positive and significant (*p* < 0.001, *r* = 0.359).

**Conclusion:**

There is a positive relationship between metacognitive beliefs with belongingness and resilience of novice nurses; nursing managers can consider educational metacognition workshops to enhance the sense of belongingness and resilience of novice nursing staff leading to improve their clinical performance in neonatal care.

## 1. Introduction

Nurses are the first to recognize the risks and critical situations of infants and take action to address them in the Neonatal Intensive Care Units [1]. Nurses are one of the largest health care providers [2], but nursing shortage has led to the use of novice nurses in the health care system, who often enter real situations early, and caused job and environmental stress and reduced their satisfaction [1, 3]. In one study, the average ratio of nurses to infants in the Neonatal Intensive Care Unit in Tehran, the capital of Iran, one nurse for more than 4 babies, was reported [1]. Novice nurses have many problems in context, including having difficulty in concentrating, lacking focused attention, and failing to maintain moment-to-moment awareness. Novice nurses in clinical settings need help and do not know what is expected of them in real settings [4, 5]. In this way, they are not able to understand their situation and solve problems effectively [6]. However, novice nurses, in order to creatively solve problems, need skills that can help them in the proper and correct use of their information [7]. The novice nurses' past experiences acquired through their academic studies include factors to enhance them, and one factor is metacognition or having the talent or skill of metacognitive ability, which helps novice nurses use these experiences in new situations [8]. Metacognition is a multifaceted concept that includes beliefs (knowledge), processes, and strategies that evaluate, monitor, or control cognition [9]. This knowledge is beliefs and theories held by people about their thinking, such as beliefs about the meaning of certain types of thoughts, the efficiency of memory, and cognitive control, and they are responsible for controlling healthy and unhealthy thoughts [10]. The results of some studies have shown that metacognitions are effective factors in the state of mental health. We can improve the situation by changing the metacognitions that increase the maladaptive ways of negative thoughts or increase the general negative beliefs [11, 12]. Thus, we can help people to structure their thinking and prepare themselves to accept their future roles [13]. Moreover, maintaining meaningful relationships with others is a permanent desire in humans, and people tend to communicate with each other for optimal performance [14].

The sense of belonging is one of the basic human needs that creates a sense of security and comfort. In general, people are trying to be accepted by others because the lack of communication with others has many cognitive, emotional, and behavioral consequences [15]. One nursing concept analysis defines the sense of belonging as the experience of a person's involvement in a system or environment so that a person feels that she is an integral part of that system or environment [16]. The absence of a sense of belonging can lead to problems such as low self-esteem and satisfaction, anxiety, depression, high level of stress, and increased tension in clinical practice [17, 18]. Belongingness is an important effect factor in workplace satisfaction, and this type of satisfaction is one of the main factors that determine nurses' resilience in difficult situation [19].

Resilience is one of the main abilities of humans enabling them to resist difficult conditions and mental pressures through effective adaptation to changes and stressful factors [20]. Warelo and Edward stated that 21st-century nurses need to skillfully develop their resilience to face professional challenges and ensure their mental health because resilience and resilient behaviors potentially help individuals to overcome negative experiences and turn these experiences into positive experiences [21].

Considering the annual employment of a large number of novice nursing staff in the medical care system and their predefined problems, it is necessary to provide solutions to improve their skills. The role of metacognition in solving problems, attitude towards problems, resilience against them, and clinical relevance in the path of gaining experiences and lack of research on the role of metacognition necessitates determining the role of each of these variables and in case of the presence of effective role to design programs to improve them. The lack of research studies that has investigated the relationship between metacognitive beliefs, belongingness, and resilience, especially in novice nurses, caused that this study was conducted to investigate the relationship between metacognition, belongingness, and resilience in novice nursing staff in Neonatal Intensive Care Units (NICUs).

## 2. Methods

The present study was a descriptive correlational study. The samples included 78 novice nursing staff working in Neonatal Intensive Care Units of three hospitals affiliated to Shiraz University of Medical Sciences. After obtaining permission from the Ethics Committee and Vice Chancellor for Research of Shiraz University of Medical Sciences, the researcher referred to the mentioned hospitals and selected the novice nursing staff who were eligible to enter the study by the purposive sampling method. It was decided that nurses with less than one year of work experience in NICU wards through the purposive sampling method entered the study. Other inclusion criteria for this study were as follows: having at least a bachelor's or master's degree in nursing or midwifery, working in the Neonatal Intensive Care Units of mentioned hospitals, and signing the informed consent form.

After obtaining informed consent from the samples, researcher provided the relevant questionnaires to novice nurses to complete. The ethics code of this research (IR.SUMS.REC.1400.217) was obtained from the Ethics Committee of Shiraz University of Medical Sciences.

In order to achieve the objectives of this study, demographic characteristic questionnaires, Wells and Hatton metacognitive beliefs, Jones Levitt belonging, and Connor–Davidson resilience questionnaires were used:

1. *Demographic Information Questionnaire.* This questionnaire contains questions such as age, sex, marital status, field of study, and type of ward; the face validity of which has been approved by several professors and experts in neonatal nursing field.
2. *Wells and Cartwright-Hatton Metacognitive Beliefs Questionnaire.* This questionnaire was designed in 2004 and has 30 questions in which each person answers four-choice questions (disagree, slightly agree, relatively agree, and strongly agree). These options are scored 1, 2, 3, and 4, respectively. This questionnaire has 5 subscales: cognitive conflict measures positive beliefs, cognitive self-awareness, uncontrollability, and the danger of thoughts and the need to control thoughts. A high score on this scale indicates a strong level of metacognition in the individual. A score between 30 and 60 indicates a weak level of metacognition, a score between 60 and 90 indicates an average level of metacognition, and a score between 90 and 120 indicates a strong level of metacognition in a person. Hatton and Wells have reported the Cronbach's alpha coefficient of this questionnaire and its components in the range of 0.72 to 0.93 and its retest reliability coefficient (with an interval of one month) of 0.73 [22]. Osoli et al. reported an internal consistency coefficient of 0.86 for this questionnaire [23]. Gharayeli and Saberi calculated the reliability of the tool using Cronbach's alpha test, which was 0.79, 0.77, 0.81, 0.80, and 0.83 for positive beliefs about worry, cognitive confidence, cognitive self-awareness, negative beliefs about the uncontrollability of thoughts, and beliefs about the need to control thoughts, respectively [24].
3. *Levett–Jones Belongingness Questionnaire.* This questionnaire was developed by Levett–Jones and Lathlean in Australia in 2008. The questionnaire has 34 items and three subscales of self-esteem, continuity, and efficiency in the Likert scale with 5 options (1 = never, 2 = rarely, 3 = sometimes, 4 = often, and 5 = always) [25]. A higher score indicates a greater degree of belongingness of the respondent to the clinical environment. The self-esteem subscale items include items 1, 3, 4, 7, 9, 10, 14, 17, 21, 23, 24, 27, and 33, and the subscale attachment includes items 8, 13, 15, 16, 25, 26, 28, 29, 30, and 34, and the subscale efficiency includes items 2, 5, 11, 18, 19, 20, 31, and 32. Items 6 and 12 do not include any subscales. The minimum and maximum scores are 32 and 160. This questionnaire has been translated and validated by Ashktorab et al. in Iran for the optimal use of other researchers. The stability of the tool was checked with the help of a test-retest, after two weeks and by completing the questionnaire by 25 nursing students and the internal consistency method using Cronbach's alpha method. The reliability of the tool was obtained by the test-retest repeatability method, *r* = 0.70. Cronbach's alpha for the whole tool was 0.90 and it was 0.88, 0.75, and 0.84 for the subscales of self-esteem, coherence, and efficiency, respectively. In the Persian version, the terms 26 and 10 were removed from the dimension of attachment and self-esteem according to the results of the factor analysis and are not included in the analysis of the results [26]. This questionnaire has been translated and validated by Mohammad et al. in Malaysia for the optimal use by other researchers. Twenty items from the original tool to measure the participants' belongingness experiences with colleagues, twelve similar items to measure the participants' belongingness experiences with other members of the health care team at work, and eight items were created through a literature review of previous studies on nursing and organization. Then, the content validity of the final questionnaire was tested and evaluated by a group of nursing personnel from two different hospitals. The revised scale has a Cronbach's alpha value of 0.93, and in further analysis, it was (0.89), (0.83), and (80/0) for 20 items on colleagues, 12 items on other members of the health care team, and 8 items on the organization [27].
4. *Connor–Davidson Resilience Scale.* This scale was developed by Connor–Davidson in 2003. This scale includes 25 items based on factor analysis; five subscales including individual competence and adequacy (8 items), tolerance of negative effects and strength against stress (7 items), positive acceptance of change (5 items), self-control (3 phrases), and spiritual effects (2 phrases) are assigned. Scoring is also based on the Likert scale between completely incorrect = 0 and always true = 5. The maximum score is 100 and the minimum score is 0. For Connor–Davidson Resilience 25 questions, Normah in Iran showed that Cronbach's alpha coefficient of all questions is 0.88. The questions have good consistency with 8 questions which were 0.81 and 0.76, respectively, and Cronbach's alpha reliability coefficients for the subscales of stubbornness, belief, and optimism were reported to be 0.60, 0.55, and 0.36, respectively [28]. Javadian and Fathi reported the reliability of the resilience scale using Cronbach's alpha method as 0.86 [29]. Regarding its validity, using the factor analysis method, the calculation of each score with the total score showed that, except for the three questions that were higher, the coefficients of the other questions were between 0.51 and 0.61 [30].

### 2.1. Ethical Considerations

Ethical approval was obtained from the Local Research Ethics Committee of Shiraz University of Medical Sciences, Iran (Approval No. IR.SUMS.REC.1400.217). In the beginning, a verbal description of the study objectives was provided for novice staff and after the assurance of the confidentiality of their information, the written informed consent was obtained from participants.

## 3. Results

From 78 novice nursing staff working in Neonatal Intensive Care Units (NICUs), 43.6% of the staff are working in internal wards of NICUs with 93.5% of the novice nursing staff aged less than 31 years. All novice nursing staff in NICUs were female (no male nurses have worked in the Neonatal Intensive Care Units during this study) and 55.1% of them are single. The majority of these staff (97.4%) had a bachelor's degree (Table 1).

The lowest score recorded for metacognition in novice nursing staff was 63 and the highest score was 125 with an average of 92.67, which indicated the high score of metacognitions. The lowest recorded score for the belongingness in novice nursing staff was 46 and the highest score was 141 with an average of 116.69, which indicated high belongingness in novice nursing staff. The lowest score of resilience in novice nursing staff was 14 and the highest score was 95 with an average of 72.87, which indicated high resilience in novice nursing staff (Table 2).

Given the quantitative nature of the two indicators, Pearson's correlation test was used to determine the relationship between metacognition and belongingness. Pearson correlation coefficient of 0.265 shows a positive relationship between metacognition and belongingness. The significance level of Table 3 (*p* < 0.05) shows the significance of this correlation coefficient at the 5% error level. In general, the results show that there is a relationship between metacognition and the belongingness of novice nursing staff in NICUs. Also, given the quantitative nature of the two indicators, Pearson's correlation test was used to determine the relationship between metacognition and resilience. Pearson correlation coefficient of 0.359 shows a positive relationship between metacognition and resilience. The significance level of the above table (*p* < 0.001) shows the significance of this correlation coefficient at the 5% error level. In general, the results show that there is a relationship between metacognition and the resilience of novice nursing staff in NICUs (Table 3).

**Figure 1:**
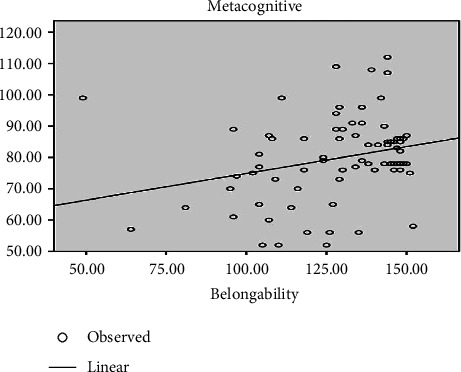
The relationship between metacognition with belongingness among novice nursing staff in NICUs.

According to the correlation coefficient (*r* = 0.265), the relationship between metacognition and belongingness is positive and significant (Figure 1).

**Figure 2:**
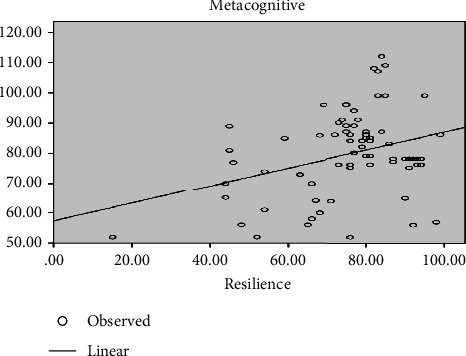
The relationship between metacognition with resilience among novice nursing staff in NICUs.

According to the correlation coefficient (*r* = 0.359), the relationship between metacognition and resilience is positive and significant (Figure 2).

## 4. Discussion

This study examined the relationship between metacognition with belongingness and resilience in novice nursing staff in Neonatal Intensive Care Units in three hospitals. The results showed that there was a significant relationship between metacognition and belongingness. The results of this research are consistent with the study of Mousavi and Alvani [31] and Chamanabad et al. [32]. In fact, nurses involved in difficult issues and problems need to simultaneously use metacognition to understand concepts, find creative solutions, and make the decision, as well as belongingness to increase clinical skills. Both of the abovementioned variables will help nurses in difficult situations; thus, the two skills will help to solve problems more favorably. Some studies have shown that cognitive skills and knowledge enable people to understand concepts, solve problems, and make decisions [33, 34]. In other words, through metacognition, novice nursing staff can solve their problems when problems arise and cause confusion. This power in solving problems and overcoming problems increases the sense of belonging as an important factor in clinical learning.

The results showed that there was a significant relationship between metacognition with resilience. In a study by Han in Korea, a significant relationship was established among metacognition, shared leadership, and resilience [35]. This study was consistent with the results of Hasani's study [36] and the study of Yoosefi and Karimipoor [37]. In explaining these findings, it can be said that belief and awareness of individual abilities and inabilities, which are considered to be part of the characteristics of the metacognition system, strengthen motivation, and initiate a series of actions to win the task. Before a person chooses a job and starts his or her efforts, she/he first collects and evaluates information about his or her capacities and abilities in that particular case. Doing this step allows him/her to consciously review themselves, whether she/he can adapt to perform a certain behavior in the face of problems or not. Also, it is also specified that how much she/he will try despite the existing problems and how long she/he will continue efforts in the specific field. In these conditions, people will achieve sufficient self-efficacy (characteristic of resilience) and will be able to cope with unpleasant or complex events more effectively, and finally, optimism and positive thinking will become active in the resilience process. By achieving resilience, people are more steadfast in their efforts, they have more confidence in their abilities, they do not drown in the waves of their doubts, they persevere in doing their tasks, and often the result of their performance is at a high level. Also, the activation of positive metacognition makes people less tense. It should be noted that the experience of tension in people causes them to engage in incompatible coping strategies (avoidance and suppression of thoughts) and the use of these strategies intensifies stress and negative emotions. This process makes people overestimate environmental threats and not have the ability to deal with problems, and this leads to a decrease in their resilience; therefore, it can be concluded that having metacognition knowledge can increase resilience.

The main limitation of this research is that the results cannot be generalized due to the small number of samples and hospitals.

## 5. Conclusion

This study showed that there is a significant relationship between metacognitive beliefs with clinical belongingness and resilience of novice nursing staff in NICU wards. In other words, the more metacognition a novice nurse has in the clinical environment, the better her/his belongingness and resilience. This also increases the belongingness to the clinical environment and increases the motivation of novice nursing staff to learn at the clinic. Therefore, it is suggested that health professionals improve the clinical belongingness and resilience of novice nursing staff in NICUs by developing training programs for novice nurses to increase the quality of care for neonates through the increasing of belongingness and resilience in nurses.

## Figures and Tables

**Table 1:** Demographic characteristics among novice nursing staff of neonatal intensive care units.

Variables	Category	Frequency	Percentage
Age (years)	20–25	26	33.3
	26–60	47	60.2
	31–35	5	6.4

Gender	Female	78	100
	Male	0	0

Marital status	Single	43	55.1
	Married	35	44.9

Education	Bachelor	76	97.4
	Master or PhD	2	2.6

Total	78	78	100

**Table 2:** The means and standard deviations of metacognition, belongingness, and resilience among novice nursing staff in NICUs.

Variables	*N*	Min. Score	Max. Score	Mean	SD
Metacognition	78	63	125	92.67	13.69
Belongingness	78	46	141	116.69	19.11
Resilience	78	14	95	72.87	14.73

**Table 3:** The relationship between metacognition with belongingness and resilience among novice nursing staff in NICUs.

Variables	Description	Belongingness	Resilience
Metacognition	Correlation coefficient	0.265^*∗*^	0.359
	Significance level	0.019	0.001
	No.		78

^*∗*^Pearson correlation coefficient test.

## Data Availability

Data used and analyzed to support the findings of this study are available from the corresponding author upon request.
